# Response Inhibition and ADHD Traits: Correlates and Heritability in a Community Sample

**DOI:** 10.1007/s10802-012-9693-9

**Published:** 2013-01-13

**Authors:** J. Crosbie, P. Arnold, A. Paterson, J. Swanson, A. Dupuis, X. Li, J. Shan, T. Goodale, C. Tam, L. J. Strug, R. J. Schachar

**Affiliations:** 1 Neurosciences and Mental Health Program, Research Institute, The Hospital for Sick Children, University of Toronto, Toronto, Canada; 2 Genetics and Genomic Biology Program, Research Institute, The Hospital for Sick Children, University of Toronto, Toronto, Canada; 3 Department of Psychiatry, Hospital for Sick Children, University of Toronto, 555 University Avenue, Toronto, ON Canada M5G 1X8; 4 Department of Pediatrics, School of Medicine, University of California Irvine, Irvine, CA USA; 5 Research Operations, Research Institute, The Hospital for Sick Children, University of Toronto, Toronto, Canada; 6 Dalla Lana School of Public Health, University of Toronto, Toronto, Canada; 7 Department of Psychiatry, Florida International University, University Park, FL USA; 8 Sackler Institute, Department of Psychiatry, Weill College of Medicine at Cornell University, Ithaca, NY USA; 9 Child Health Evaluative Sciences, Research Institute, The Hospital for Sick Children, University of Toronto, Toronto, Canada

**Keywords:** ADHD, Stop signal task, General population, Endophenotype, Heritability

## Abstract

Endophenotypes or intermediate phenotypes are of great interest in neuropsychiatric genetics because of their potential for facilitating gene discovery. We evaluated response inhibition, latency and variability measures derived from the stop task as endophenotypes of ADHD by testing whether they were related to ADHD traits in the general population, heritable and shared genetic risk with ADHD traits. Participants were 16,099 children and adolescents, ages 6 to 18 years who visited a local science center. We measured ADHD traits using the Strengths and Weaknesses of ADHD-symptoms and Normal-Behavior (SWAN) rating scale and performance on the stop signal task (SST)—response inhibition (SSRT), response latency (GoRT), and response variability (GoRTSD). Regression analysis was used to assess the relationship of cognitive measures and ADHD traits while controlling for family, age, sex, ethnicity, socioeconomic status and treatment status. Heritability of ADHD and cognitive traits was estimated using SOLAR in 7,483 siblings from 3,507 families that included multiple siblings. Bivariate relationships between pairs of variables were examined. Individuals with greater ADHD trait scores had worse response inhibition, slower response latency, and greater variability. Younger participants and girls had inferior performance although the gender effects were minimal and evident in youngest participants. Inhibition, latency, variability, total ADHD traits, inattention and hyperactivity-impulsivity scores were significantly heritable. ADHD traits and inhibition, but not latency or variability were coheritable. In the largest study in the general population, we found support for the validity of response inhibition as an endophenotype of ADHD.

**Electronic supplementary material:**

The online version of this article (doi:10.1007/s10802-012-9693-9) contains supplementary material, which is available to authorized users.

Attention deficit hyperactivity disorder (ADHD) is a complex disorder characterized by extremes of inattention, hyperactivity and impulsivity, by clinical and genetic heterogeneity and by high heritability whether it is measured as a discrete diagnostic entity or as a continuous trait in the general population (Swanson et al. 2009; Thapar et al. 2007). Despite high heritability, no gene conferring large relative risk has been identified. Meta-analyses of ADHD linkage studies supports genome-wide significance on 16q21-16q24 (Zhou et al. 2008) but the largest and most comprehensive genome-wide association study did not identify any genome-wide significant findings (Franke et al. 2009; Neale et al. 2010). Identification of the genetic contributions to the disorder is complicated by phenotypic and genetic heterogeneity, low penetrance and low statistical power of existing studies (van der Sluis et al. 2010). One way to enhance the power of genetic discovery is to reduce clinical and genetic heterogeneity by use of endophenotypes (Kendler and Neale 2010). Endophenotypes are biological traits that mediate the association between some of the genetic risks for a disease and the disease phenotype and which have a genetic architecture that is less complex than that of the disorder itself (Crosbie et al. 2008; Gottesman and Gould 2003; Szatmari et al. 2007). Potentially useful endophenotypes are those that are associated with the disorder, evident in relatives because of their increased genetic risk, heritable and share genetic risk with the disease phenotype (Crosbie et al. 2008; Gottesman and Gould 2003; Kendler and Neale 2010).

Considerable research into ADHD endophenotypes has focused on executive function processes that are involved in planning, decision making and action. Among the most extensively studied candidate endophenotypes are motor response inhibition, response latency and response variability (Frazier-Wood et al. 2012). Meta-analysis indicates that patients with a diagnosis of ADHD have deficient inhibitory control compared with controls with a medium effect size of 0.63 (Lipszyc and Schachar 2010) and that inhibitory control is worse in non-ADHD siblings and in parents of ADHD probands than it is in normally developing individuals (Goos et al. 2009; Schachar et al. 2005). Twin studies indicate that inhibitory control is heritable (Friedman et al. 2008; Schachar et al. 2011). Several risk alleles are associated with poor inhibition (Bellgrove and Mattingley 2008; Langley et al. 2004; Swanson et al. 2000) and with brain activity associated with response inhibition (Barnes et al. 2011; Cummins et al. 2011). Response speed and variability reflect consistency of attention and effort. Meta-analysis shows a small-to-medium effect size for response latency and a medium effect size for variability (0.71) (Lipszyc and Schachar 2010; Sergeant 2000) in distinguishing individuals with and without an ADHD diagnosis. Twin studies have found that response variability was heritable (Frazier-Wood et al. 2012; Friedman et al. 2008; Young et al. 2009).

Endophenotypes derived from simple laboratory tests such as the SST have the potential advantage of being suitable for general population research where they could help identify individuals at genetic risk for disorder without a time-consuming and costly diagnostic assessment. Despite this potential utility, there has been little research of validity of candidate endophenotypes in the general population (c.f. Cornish et al. 2005). In this study, we use the stop signal task (SST) (Logan et al. 1997; Verbruggen and Logan 2009a) to investigate the relationship of response inhibition, latency and variability and ADHD traits in the general population. If response inhibition, latency and variability are genetically informative endophenotypes, they should be correlated with ADHD traits even after controlling for age, gender, socioeconomic status and ethnicity. They should also be heritable and share genetic risk with ADHD traits. We investigated these questions using the SST and the Strengths and Weaknesses of ADHD-symptoms and Normal-Behavior (SWAN) rating scale as a measure of ADHD traits. In related individuals, we used Sequential Oligogenic Linkage Analysis Routines (SOLAR) to estimate heritability of individual traits and shared genetic risk of pairs of traits.

## Method

### Participants

We evaluated 16,099 individuals, 6 to 18 years of age, at the Ontario Science Centre (OSC) in Toronto, Canada (http://www.ontariosciencecentre.ca). The OSC has about one million visitors each year of all ages, social backgrounds and ethnic groups and is a leading developer of interactive exhibitions for science centres around the world. Participants were tested at workstations with dividers in a specifically designed exhibition on genes, brain and behaviour. Visitors that showed interest in the display were invited to participate. Typically, there are several such participatory research studies going on at the Science Centre at any one time as part of the mission of the Centre. Participants were individually supervised during testing by undergraduate university students.

Parents provided consent and SWAN ratings for children ages 6–12 while children completed the SST. Individuals aged 13–18 provided consent and self-report SWAN ratings before completing the SST. Participants provided a saliva sample for genetic analyses. The study was approved by The Hospital for Sick Children’s research ethics board. Testing was restricted to 30 min. No compensation was given to participants.

Socioeconomic status as measured by household income was estimated from postal codes referenced against 2006 Canadian national census data, which provides size-adjusted household income for neighbourhoods (Wilkins 2009). Ethnicity was estimated using a self-report questionnaire and participants were classified as Caucasian if four grandparents were Caucasian. Caucasians made up the majority of cases; the remainder were coded as non-Caucasian for the purpose of this study (although detailed ethnicity information was available). There were 991 participants who reported a prior diagnosis of ADHD (6.2 %) of which 395 reported taking ADHD medication within the previous 2 days and 19 who didn’t report a diagnosis of ADHD but did report taking ADHD medication (0.1 %).

### ADHD Trait Measure

The SWAN is based on the 18 ADHD items in the DSM-IV (American Psychiatric Association 1994; Swanson et al. 2001). Items are worded to capture both strengths and weaknesses on a seven point scale (+3 = far above average indicating a strength in a particular trait or low ADHD trait scores, to −3 = far below average indicating the presence of an ADHD trait) (Swanson et al. 2001). Scores for the nine inattention and for the nine hyperactivity-impulsivity trait items were also calculated. There is a high correlation between Conners Parent and Teacher Rating Scales (Conners 1998) and SWAN ratings of approximately 0.90 for teacher ratings and 0.80 for parent ratings (Cornish et al. 2005) and there is high agreement on which individuals were in the extremes of the distribution on these two measures (Polderman et al. 2007). For clarity of interpretation and discussion, SWAN scores were reversed in the analyses such that higher SWAN scores represented higher ADHD traits. The paper will refer to these scores as high or low ADHD traits; when actual values are presented, positive values represented greater ADHD traits (+54 indicating the highest possible ADHD trait score, and −54 representing the lowest possible ADHD trait score).

### Stop Signal Task

Response inhibition, latency and response variability were measured using the SST (Logan et al. 1997). The SST consists of two concurrent tasks—a go and a stop task. The go task is the presentation of one of two letters (an X or an O) on each trial. Participants were required to make a response to the go task stimuli as quickly and as accurately as possible by pressing one key of a hand held game pad for an X and the other for an O. The stop task involved an auditory signal which was presented, at random, on 25 % of trials. The stop signal instructed participants to withhold their response on that particular trial. The stop tone was a 1,000 Hz tone emitted by the computer and presented by headphones at a comfortable listening level. The task consisted of a practice block (24 trials; 18 go trials; six stop trials) and four experimental blocks of 24 trials for a total of 72 go trials and 24 stop trials. Each trial began with a fixate stimulus which was presented for 500 ms followed by the go-signal (X or O) which was presented for 1,000 ms. The total trial duration was 3,500 ms allowing 3,000 ms for a go task response. The task paused briefly after each block so that the supervisor could check if there were any error messages indicating that the participant was not following task instructions.

Performance in the stop signal task can be modeled as a race between two independent processes—the response execution process initiated by the presentation of the go signal and finishing with the motor response and the stop process initiated by the presentation of the stop signal (Verbruggen and Logan 2009b). If the stop process finishes before the go process, the response is stopped. If the go process finishes before the stop process, the response is executed just as if no stop signal were presented. The outcome of the race and the probability of stopping a particular response depend on the speed of go response and the speed of the internally generated stopping process the latency of which is known as stop signal reaction time (SSRT). The outcome of the race also depends on the delay between the onset of the go and the onset of the stop processes. Delay is under experimental control. Stop signal delay, initially set at 250 ms, was dynamically adjusted depending on performance. If participants were able to stop on a particular trial, the delay was increased by 50 ms in order to make it more difficult to stop on the next trial. If they were unable to stop, the delay was shortened by 50 ms (Logan et al. 1997). Go reaction time and variability is evident in the latency of trials that do not involve a stop signal. The primary dependent variable, SSRT can be estimated by subtracting mean delay from mean go response time on no-signal trials when the participant’s probability of stopping is approximately 0.50. With departures from probability of inhibition given a stop signal from 0.50, SSRT can be calculated through an integration procedure as was done in the current study. Go reaction times in which no stop signal were presented were rank ordered and the go reaction time that corresponded to the probability of inhibition was determined. For example, if a participant inhibited 60 % of their go responses, one finds the 60th slowest go reaction time. All slower go responses would have been stopped; all faster ones would have been executed. Interpolated SSRT is estimated by subtracting mean delay from the integrated go reaction time (Verbruggen and Logan 2009a) and is the primary dependent variable in this study.

Participants (970) were excluded if they performed the task in an invalid manner (go task with less than 66 % accuracy, mean GoRT of less than 100 ms indicating that they were guessing on the go trials, or inhibited on more than 80 % or fewer than 20 % of stop signal trials). As an added check, we recalculated all models on participants who inhibited more than 0.40 or less than 0.60 (95 % confidence interval for probability of inhibition of 0.50). We present models for the entire sample because results were not affected by this additional set of exclusion criteria. Participants (147) were also excluded if they interrupted the task before completion because of time constraints on their family visit to the Science Centre or pushed the wrong buttons on the game pad device used to collect responses (180).

## Analyses

Regression analysis was conducted to identify the best combination of variables making a unique contribution to SSRT, GoRT and GoRTSD while considering family as a random effect (Singer 1998). In order to normalize the residuals from each model, we modeled the log of the stop task variables. Because of the high correlation of latency and variability, we calculated a new variable, GoRTSD/GoRT. However, the distribution of the residuals violated the assumptions of the model and no transformation of the derived variable improved the result. Therefore, we chose to control GoRT in models of GoRTSD by entering GoRT into regression models. Models included gender, ADHD traits and age which were added using a forward stepwise approach. The higher order terms, age*age, gender*age, age*age*gender were also assessed when main effects were significant. We plotted predicted values obtained from models with and without the higher order terms in order to compare the predicted values between the simpler and more complex models to assess the clinical significance of the higher order terms.

In order to evaluate the effect of age, gender, and ADHD trait on the stop task values, we calculated an approximation of Cohen’s effect size using the equation

$$ {{{\left( {\mathrm{pred}1-\mathrm{pred}2} \right)}} \left/ {{\mathrm{sqrt}\left( {\mathrm{stderrpred}1\hat{} 2-\mathrm{stderrpred}2\hat{} 2} \right)}} \right.} $$

where stderrpred1 and stderrpred2 are the standard deviations of a single observation as obtained from the regression model. Effect sizes were calculated for specific scenarios, such as the effect of gender at age 6 in children with a Swan score of 54.

To determine if extremely low SWAN scores might also be maladaptive, we compared participants with high, medium and low SWAN scores on rates of parent-reported ADHD, anxiety, depression, learning disability and other disorders.

We estimated SSRT for 414 (2.6 %) participants who reported taking medication for ADHD within 48 h of testing by adjusting SSRT by 0.75 effect size based on the results of published studies of the effect of a moderate dose of stimulant medication on SST performance (Tannock et al. 1995). Results did not differ when analyses were conducted with and without these participants; consequently they were excluded from the subsequent analyses. The final sample (14,388) consisted of 7,176 boys (49.9 %) and 7,212 girls (50.1 %).

Heritability of each SST variable and of ADHD traits with age and sex as covariates was estimated using SOLAR (Almasy and Blangero 1998) in the subset of 3,507 families that included multiple full siblings (7,483). Of these families, 3,081 were from families with two children or adolescents, 387 with 3, 35 with 4, and 4 with 5. SOLAR decomposes the total variance of the phenotype into components that are the result of genetic effects (i.e. polygenic, additive genetic variance), measured covariates and random environmental effects (i.e. measured environmental factors and random unmeasured factors). The relative contribution of genetic factors to each trait is then estimated by heritability (h^2^): the ratio of the genetic variance component to the residual (after removal of variance explained by covariates) phenotypic variance. We calculated confidence intervals for heritability estimates under the asymptotic normality assumption of the Maximum Likelihood estimator where sample size is ~ 7,000 using the formula

$$ 100\left( {1-\alpha } \right)\%\;\mathrm{asymptotic}\;\mathrm{confidence} \operatorname {int}\mathrm{ervals}\;\left[ {{{\mathrm{h}}^2}-\mathrm{Z}\left( {{\alpha \left/ {2} \right.}} \right)*\mathrm{SE},\;{{\mathrm{h}}^2}+\mathrm{Z}\left( {{\alpha \left/ {2} \right.}} \right)*\mathrm{SE}} \right] $$

(Neale and Miller 1997).

Bivariate analyses were conducted on pairs of neuropsychological measures that demonstrated significant univariate heritability to partition the phenotypic correlation between two traits (RhoP) into genetic (Rhog) and environmental (Rhoe) correlations according to the equation

$$ RhoP=Rhog\sqrt{{h_1^2}}\sqrt{{h_2^2}}+Rhoe\sqrt{{\left( {1-h_1^2} \right)}}\sqrt{{\left( {1-h_2^2} \right)}} $$

where h_1_^2^ and h_2_^2^ correspond to heritabilities of traits 1 and 2, respectively. Evidence of pleiotropy (a common set of genetic influences affecting more than one trait) is indicated by a genetic correlation significantly different from zero (Rhog = 0: no shared gene effect; Rhog = 1 or –1: complete pleiotropy). We estimated heritability of SST variables with and without SWAN ADHD traits as covariates in order to control for the influences of ADHD traits on cognitive performance. Heritability estimates were essentially unchanged and are presented with ADHD controlled.

## Results

### Sample Characteristics

**Fig. 1:**
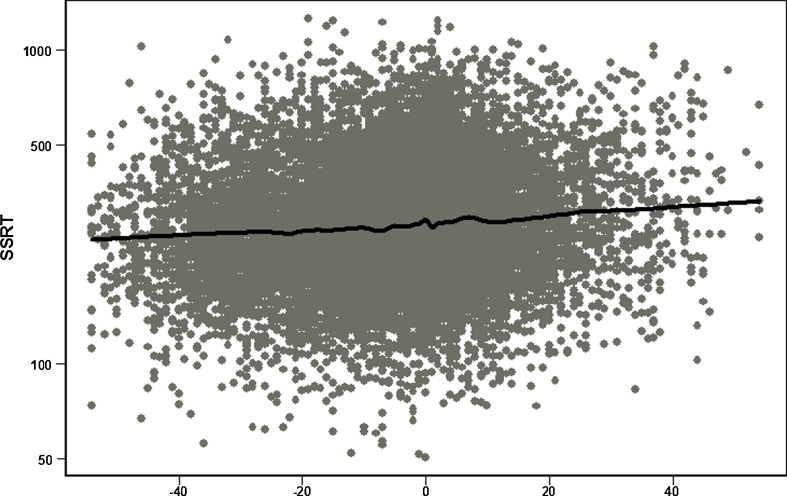
Scatterplot of response inhibition (SSRT; interpolated and plotted on log scale) and ADHD traits (SWAN scores) for entire sample of 14,388 participants of all ages and both sexes

**Fig. 2:**
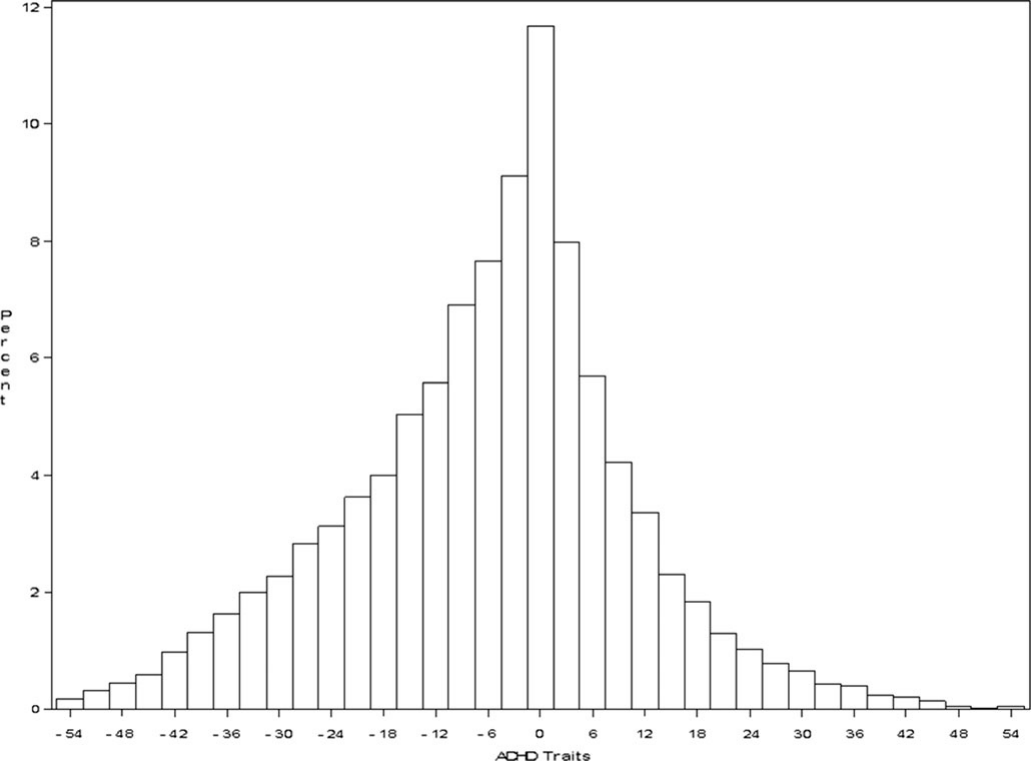
Distribution of ADHD traits in general population sample. Note: ADHD traits were measured using the SWAN. The SWAN scores were reversed such that higher ADHD traits were represented by higher SWAN scores

Table 1 presents mean SWAN scores for ADHD traits, age, sex and ethnicity for the final study sample (N = 14,388) and for 1,711 cases who were excluded because they had taken medication prior to participation (414) or who had invalid or incomplete performance the SST (1297). Compared with included cases, excluded cases had significantly higher (i.e. more severe) ADHD traits and were more often younger males, but did not differ in ethnicity or socioeconomic status. Half of the sample was Caucasian. Figure 1 shows a scatterplot of response inhibition and ADHD traits. Figure 2 shows the distribution of ADHD traits in the sample. The final sample had a mean SSRT of 304.4 ms (SD = 145.6), GoRT of 599.3 ms (SD = 111.1) and GoRTSD of 158.7 ms (SD = 55.6).

**Table 1:** Characteristics (mean or %) of study sample (*N* = 14,388) and excluded cases (*N* = 1,710)

Variable	Study sample (*n* = 14,388)	Excluded cases (*n* = 1,711^a^)	Significance of group difference
	Mean (SD)	Mean (SD)	
ADHD traits	−5.8^b^ (16.3)	3.2 (18.9)	<0.0001
Age	11.2 (2.8)	10.1 (2.8)	<0.0001
Male	49.9	57.2	<0.0001
ADHD	3.7	26.8	<0.0001
Caucasians	51.3	52.0	0.50

^a^1711 participants had taken medication within 2 days of participation in the study (414) or had invalid or incomplete performance on the SST (1297) as defined in the Method section

^b^ADHD traits (SWAN scores) where higher scores indicate greater severity of ADHD traits (see text for details)

There was a bias toward more economically privileged participants. Approximately 16 % of study participants were from the poorest neighbourhoods compared with 37 % in the surrounding metropolitan area, whereas 25 % of participants were from the richest neighbourhoods compared with 16 % in the surrounding area. Neither ethnicity nor socioeconomic status had a significant effect on the ADHD traits or SST variables or interactions and are not considered further. Rates of reported psychiatric disorder were lowest for individuals with lowest ADHD trait scores and increased with increasing ADHD trait scores suggesting that extremely low scores were not maladaptive (data available).

### Stop Signal Task Performance and Trait-ADHD

Supplementary Figures 1–3 show the distribution of SSRT, GoRT and GoRTSD prior to transformation. Q-Q plots for the models after transformation (Supplementary Figures 4–6) showed approximately normally distributed residuals indicating that the models were correctly specified, there was generally good model fit and the models were not highly influenced by outliers.

Gender (*p* = 0.0002), ADHD traits (*p* < 0.0001), age (*p* < 0.0001), age*gender (*p* = 0.02) and age*age (*p* < 0.0001) were significantly associated with SSRT scores. Children with higher ADHD trait scores had longer SSRT representing inferior inhibition, as did girls, and younger individuals. There was a significant gender effect on SSRT across all levels of ADHD traits at age 6, with the largest difference (36 ms) seen at an ADHD trait score of 54 (Table 2). This difference corresponded to an effect size of 0.34. There was no significant gender effect at age 18. The effect of ADHD trait score on SSRT was seen across each age and gender group, with the highest difference observed in females at age 6, where the difference of 138 ms seen between the predicted SSRT at a low ADHD trait score of −54 and a high ADHD trait score of 54 corresponded to an effect size of 1.48.

**Table 2:** Stop task values (95%ile confidence intervals) estimated from the model, by gender, ADHD trait score and age extremes

ADHD Trait^a^		Female		Male	
		Age 6	Age 18	Age 6	Age 18
SSRT	Lowest (−54)	398 (385; 412)	177 (170; 183)	371 (359; 384)	177 (170; 185)
	Highest (54)	536 (516; 557)	238 (229; 247)	500 (483; 518)	239 (228; 249)
GoRT	Lowest (−54)	735 (723; 747)	521 (512; 530)	693 (682; 705)	528 (517; 539)
	Highest (54)	765 (752; 779)	542 (532; 552)	722 (710; 734)	549 (537; 561)
GoRTSD	Lowest (−54)	205 (200; 210)	118 (115; 121)	188 (183; 193)	119 (115; 122)
	Highest (54)	256 (249; 264)	147 (143; 152)	235 (229; 241)	148 (143; 153)

*SSRT* stop signal reaction time; *GoRT* mean response latency; *GoRTSD* response variability

^a^Negative values of the ADHD trait indicate lower ADHD trait values and positive values indicate higher ADHD trait values

Gender (*p* < 0.0001), ADHD traits (*p* < 0.0001), age (*p* < 0.0001), age*age (*p* < 0.0001), age*gender (*p* < 0.0001) and age*age*gender (*p* = 0.0005) were significantly associated with GoRT. On average, boys, older children and those with lower ADHD trait scores had faster response latencies. ADHD traits (*p* < 0.0001), gender (*p* < 0.0001), age (*p* < 0.0001), age*age (*p* < 0.0001) and age*gender (*p* < 0.0001) significantly predicted GoRTSD. Models of GoRTSD that included GoRT did not change estimates of these effects. On average, individuals with lower ADHD trait scores, boys and older individuals were less variable than girls, younger participants and those with more ADHD traits. The largest difference in GoRT between the lowest and highest ADHD trait score of 30 ms (ES = 0.35) was seen among females at age 6; however, differences were similar across other genders and age combinations. The difference of 51 ms seen on the GoRTSD between an ADHD trait score of −54 and 54 for females at age 6 corresponded to an effect size of 1.4. As with SSRT, the gender effects for GoRT and GoRTSD were greater at age 6 with a difference of 43 ms (ES = 0.50) on the GoRT and a difference of 21 ms (ES = 0.60) on the GoRTSD (Table 2). There was no significant gender effect at age 18 on either measures.

Models that included ADHD traits*ADHD traits, age*age and age*age*gender did not significantly improve prediction of SSRT, GoRT or GoRTSD. Inspection of plots indicated that the relationship of Swan ADHD trait scores and each of the stop task variables did not vary perceptibly across different levels of the Swan. ADHD inattention and ADHD hyperactivity-impulsivity were related to SSRT, GoRT and GoRTSD in the same way as ADHD trait total scores.

### Heritability Estimates for Stop Signal Task Variables

Univariate heritability (estimates, confidence intervals) adjusted for age and sex were significant for SSRT [h^2^ = 0.31 (0.03), p = 6.3 × 10^−23^], GoRT [h^2^ = 0.26 (0.03), p < 9.71 × 10^−16^] and GoRTSD [h^2^ = 0.28 (0.03), p < 3.9 × 10^−17^]. Total ADHD trait scores [h^2^ = 0.38 (0.03), p < 2.7 × 10^−36^], hyperactivity-impulsivity [h^2^ = 0.4 (0.03), p < 4.7 × 10^−40^] and inattention [h^2^ = 0.24 (0.03), p < 3.4 × 10^−15^] were also heritable. Age and sex accounted for differing proportions of phenotypic variance, but had little effect on estimates of heritability. Exclusion of ADHD trait scores in the heritability estimates had no impact on heritability estimates.

Table 3 shows bivariate heritability estimates for ADHD traits, SSRT, GoRT and GoRTSD adjusted for age and sex. ADHD traits and SSRT showed significant genetic correlation (0.18), indicating modest shared genetic risk for greater ADHD traits and longer SSRT. ADHD traits and GoRT and GoRTSD did not show significant bivariate heritability. Inattention and hyperactivity-impulsivity had a genetic correlation of 1 indicating complete pleiotropy. There was a significant genetic correlation of SSRT and inattentive (0.23) and of SSRT and hyperactive-impulsive (0.14) traits. Hyperactivity-impulsivity, but not inattention scores were genetically correlated with GoRT (−0.19). There was evidence of substantial co-heritability of GoRT and GoRTSD (0.71) and for SSRT and GoRTSD (0.42), but not SSRT and GoRT (−0.11). Inclusion of cases who reported taking medication for ADHD within 48 h of participation did not alter substantially the results of the analyses.

**Table 3:** Results of bivariate heritability analyses: genetic correlations (standard errors) among neurocognitive measures^a^

	Rhog^b^ (S.E) ADHD trait	ADHD_IA	ADHD_HI	SSRT	GoRT	GoRTSD
SSRT	0.18 (0.07)^c^	0.23 (0.08)^c^	0.14 (0.07)^c^	1	−0.11 (0.08)	0.42 (0.08)^c^
GoRT	−0.12 (0.07)	−0.04 (0.09)	−0.19 (0.07)^c^		1	0.71 (0.05)^c^
GoRTSD	0.05 (0.07)	0.09 (0.09)	0.02 (0.07)			1

*SSRT* stop signal reaction time; *GoRT* mean response latency; *GoRTSD* response variability; *ADHD*_*IA* SWAN inattentive subscore; *ADHD*_*HI* SWAN hyperactive-impulsive subscore

^a^all SOLAR models include age, sex, and ethnicity as covariates

^b^Evidence of pleiotropy is indicated by a genetic correlation significantly different from zero (Rhog = 0: no shared gene effect; Rhog = 1 or −1: complete pleiotropy)

^c^significant *p*-value tests indicating genetic correlation

## Discussion

In the largest general population study reported to date we found support for the validity of response inhibition as an endophenotype of ADHD. Response inhibition, latency and variability were significantly correlated with ADHD traits even after controlling for age and gender. Analysis of the family data indicated that inhibition, latency and variability were heritable; however, of these three measures only inhibition shared genetic risk with ADHD traits. Current results have implications for understanding the genetic contributions to ADHD traits and to higher-order executive functions as well as for the design of genetic studies.

Participants with greater ADHD traits had longer SSRTs indicating inferior inhibition, slower response latencies and greater response variability than did those with fewer ADHD traits. The relationship of ADHD traits and inhibition was strong. Participants with highest ADHD trait scores had SSRTs that were substantially slower indicating less efficient inhibition than among those with the lowest trait scores across age and sex. The magnitude of these differences was substantial for both girls (e.g., age 6; 138 ms, ES = 1.48) and boys (e.g., age 6; 129 ms, ES = 1.48). These effects are comparable to those found in a meta-analysis of studies comparing ADHD clinic cases and healthy controls (Lipszyc and Schachar 2010). In that review, ADHD cases had a mean SSRT of 330 ms and controls had a mean score of 254 ms. Participants in those studies were approximately 10–12 years of age. Estimates of SSRT in the current data for children of that age were 329 ms for those with greatest ADHD traits scores and 231 ms for those with lowest ADHD trait scores. These converging data provide considerable support for the current results. We conclude that this general population sample included individuals at the extremes of ADHD trait and SST performance distributions that are equivalent to cases and controls typical of clinic-based case–control studies. Indeed, we found that 6.3 % of all participants self-reported a diagnosis of or treatment for ADHD supporting the conclusion that the sample was quite representative of the general population. Participants with high ADHD trait scores were substantially more variable than those with low ADHD trait scores with effect sizes that are comparable to those observed for response time variability in previous studies (Frazier-Wood et al. 2012). There was far less of a difference in response latency between those with high and those with low ADHD trait scores. We also note that the relationship of ADHD traits and each cognitive measure was consistent across the entire distribution of ADHD indicating that associations were essentially linear and not driven by those at the extremes of the ADHD trait.

Univariate heritability estimates were significant for SSRT, GoRT, and GoRTSD. The moderate magnitudes of these heritability estimates (from 0.26 to 0.31) are comparable to heritability estimates from prior studies of executive functions, specifically those on SSRT (Andreou et al. 2007; Frazier-Wood et al. 2012; Friedman et al. 2008; Kuntsi et al. 2010; Schachar et al. 2011). Moreover, SSRT, GoRT and GoRTSD were heritable even with adjustment for the well-documented effect of ADHD traits on these parameters indicating that heritability of cognitive traits are similar across the range of ADHD traits. We interpret the observed interfamilial correlation as indicative of genetic effects because previous twin studies show that familial influences on ADHD traits (Faraone and Doyle 2000) and on SST parameters (Schachar et al. 2011) are largely due to genetic effects with minimal influence of shared environment.

There was evidence of shared genetic risk between poor response inhibition (longer SSRT) and high ADHD trait scores whereas there was little evidence for shared genetic risk for ADHD traits and either response latency (GoRT) or variability (GoRTSD) (c.f. Kuntsi et al. 2010; Frazier-Wood et al. 2012). Shared genetic risk implies the potential for common genetic contributors. The significant although modest phenotypic and genetic correlation of SSRT and SWAN scores indicates that the ADHD phenotype is complex and is likely to be the final common outcome of multiple biological pathways only some of which involve deficient motor response inhibition. Significant genetic correlation indicates that genetic factors influence the extent to which these traits overlap. However, significant genetic correlation does not prove that a purported endophenotype has a simpler genetic architecture. Ultimately, the utility of an endophenotype will be proven in gene discovery. The role of this paper was to assess the potentially utility of response inhibition, latency and variability as measured in the SST as candidate endophenotypes.

The results of the current study are consistent with previous research on the link between ADHD and deficient response inhibition, latency and variability in clinical samples. Current results also confirm the findings of the two studies that have been conducted in the general population; Cornish et al. (2005) in an unselected general population sample studied with a different measure of inhibition and Friedman et al. (2008) in a twin sample studied with the SST. In the later sample as in the current study, there was evidence of shared genetic risk between a composite measure of inhibitory control and impulsiveness (Young et al. 2009).

Older participants exhibited better response inhibition than did younger participants as has been found in most (Williams et al. 1999) but not all (Friedman et al. 2008; Young et al. 2009) studies of inhibition and other executive functions. Older participants were also faster and less variable. Males were significantly although minimally better inhibitors and were faster and less variable in their responses than were girls, but the male advantage was not evident in older participants. There was no interaction between gender and ADHD trait scores indicating that gender effects were similar for those with high and low ADHD traits. Neither ethnicity nor socioeconomic status affected SST performance significantly. Consistent with prevalence rates for the disorder, ADHD traits were more marked among males. In contrast to the supposed increase prevalence of inattentive subtype of ADHD in females, we found that males had higher scores for both inattention and hyperactivity-impulsivity as measured by the SWAN.

In contrast to the results of twin studies, age had no significant effect on heritability of the cognitive measures studied here (c.f. Friedman et al. 2008). The heritability of ADHD traits as measured by the SWAN is comparable to some (Hay et al. 2007) but not other (Polderman et al. 2007) twin studies and is lower than typically reported using other measures of ADHD. Hay et al. (2007) speculated that the increased rating options available in the SWAN afford a greater opportunity than other rating scales for informants to rate their siblings as similar.

The study revealed details of heritabilities of ADHD subcomponents. Both inattention and hyperactivity-impulsivity were associated with inhibition, latency and variability. Both dimensions were heritable even after controlling for age and sex and the two trait dimensions shared substantial genetic risk as has been found in previous twin studies (Hay et al. 2007; Polderman et al. 2007; Swanson et al. 2001). Both dimensions share genetic risk with response inhibition. The genetic risks that are shared by hyperactivity-impulsivity and response latency seem to increase the behavioral trait and decrease response latency.

One limitation of evaluating executive function in the general population is that many participants took medication for ADHD within 48 h of participation in the study. On one hand, these observations support the conclusion that the community sample was typical of the population thereby enhancing the generality of our observations and conclusions. On the other hand, stimulants are known to improve response inhibition (Tannock et al. 1995) raising the possibility that our data could be censored at the high end of the distribution. The problem of studying disorders in the general population with a substantial proportion under treatment for the disorder of interest is common to many disorders (Tobin et al. 2005). The effect of therapy in epidemiological studies could distort trait scores causing reduction in the estimated effect of etiological determinants and a marked loss in statistical power. Various strategies have been used to correct for this effect such as exclusion of treated individuals and use of treatment as a covariate. We addressed this potential confound with what is thought to be the optimal strategy, namely by estimating the effect of stimulant treatment on inhibition from a prior study in order to introduce a correction among medicated individuals, thereby reducing the bias arising from treatment or from their exclusion (Rice et al. 2009; Tobin et al. 2005). We applied this correction for medication effect to the scores of the 414 medicated participants based on published studies that showed a 0.75 effect size effect of stimulant medication (Tannock et al. 1995). Multivariate model parameters and heritability estimates were unaltered when models were rerun following this correction. This type of correction allows for the inclusion of treated participants in genetic studies.

Although study of individuals in the general population is a powerful strategy for the cost-effective collection of samples for cognitive and genetic research, it is important to note that it is far more difficult to collect data on multiple cognitive measures in this setting. Friedman et al. (2008) has argued that combining multiple cognitive measures into a latent factor can increase the heritability of clusters of executive control measures by minimizing the effect of variance not attributable to the particular executive control construct. They argued that latent factor scores might be even more powerful in genetic studies than any single measure.

Another limitation of using cognitive tasks to measure ADHD endophenotypes is evident in the participants who had to be excluded because of invalid stop task performance. Many of these participants had invalid performance because of administration errors such as pushing the wrong buttons on the game pad device used to collect responses or leaving the task before it was complete because their family wished to continue their visit to the Science Centre. These errors may have been minimized through more intensive participant supervision, although it is unlikely that they can be eliminated totally in a busy general population setting. However, a portion of invalid stop task data is non-random. Individuals with invalid performance had significantly higher ADHD trait scores suggesting a systematic bias, which would serve to exclude those participants of greatest interest. These participants were also younger and more likely to be males. Given knowledge of participants’ age, gender and ADHD trait scores, it would be possible to estimate their performance using a regression model. Although the distribution of household income among Science Centre visitors was skewed toward higher incomes compared to the surrounding community, social class did not make a significant contribution to models of inhibition, latency, or variability or to ADHD traits.

The current results support the strategy of using endophenotypes in a general population for selection of individuals at high and low genetic risk for ADHD for genetic study. The “extreme trait” (also known as “selective genotyping”) approach using endophenotypes is a particularly powerful and cost-effective design for detecting genetic association (Huang and Lin 2007; Van Gestel et al. 2000). The approach has been successful in studies of QT interval (Arking et al. 2006), obesity (Herbert et al. 2006), foetal haemoglobin level (Menzel and Thein 2009), and in one previous candidate gene study for ADHD traits (Cornish et al. 2005). Another advantage of this approach is increased ease of collection due to the ready availability of general population samples compared to clinically ascertained subjects. We estimated that the cost of accruing participants from the general population was 10 % of the per subject cost in clinic samples.

Based on the current results and on previously published research, we conclude that inhibition is a heritable trait which is sensitive to variation across the range of ADHD traits and shares genetic risk with ADHD traits. Identification of genes involved in risk for inhibition may be a powerful way to identify ADHD related genetic risks.

## Electronic supplementary material

Below is the link to the electronic supplementary material.

ESM 1 (PDF 42.9 KB)
