# Supplementary material for: Response Inhibition and ADHD Traits: Correlates and Heritability in a Community Sample
Source: J Abnorm Child Psychol. 2013 Jan 13;41(3):497–507. doi: 10.1007/s10802-012-9693-9 (PMC3600128; doi:10.1007/s10802-012-9693-9)

Supplementary Figure 1 Distribution of SSRT in sample

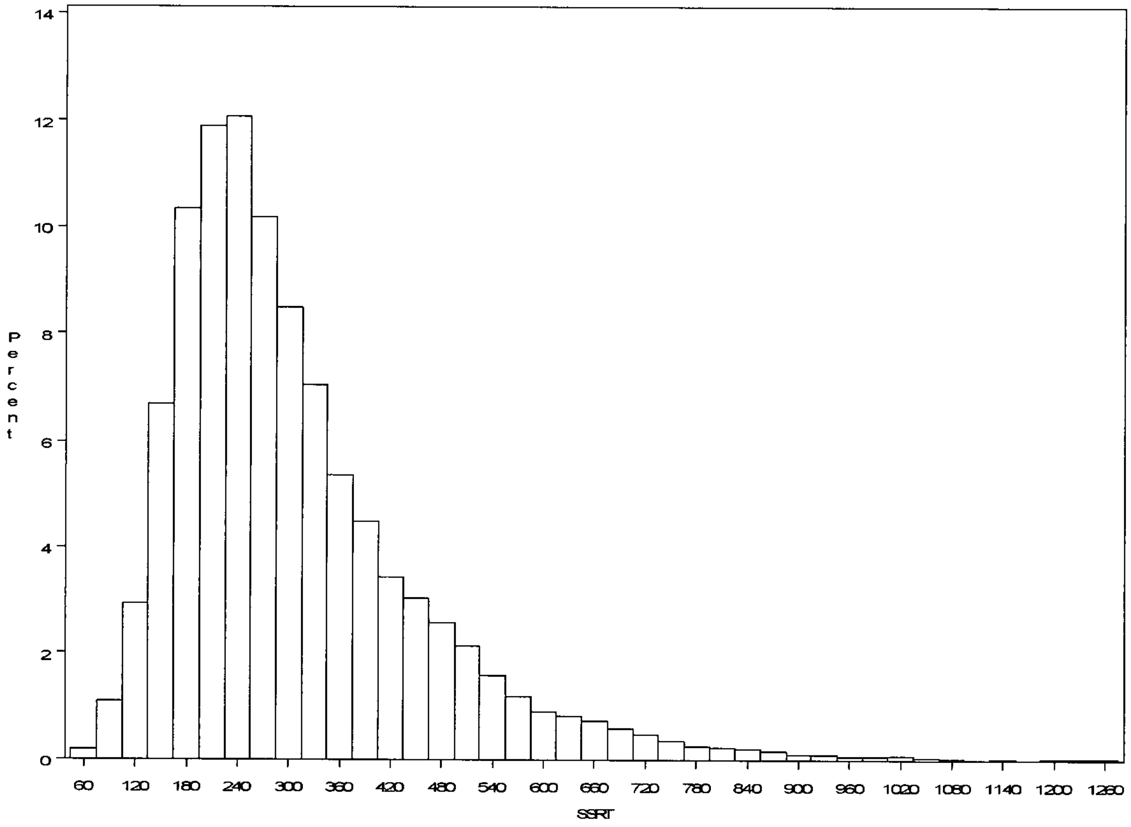

Supplementary Figure 2 Distribution of GoRT in sample

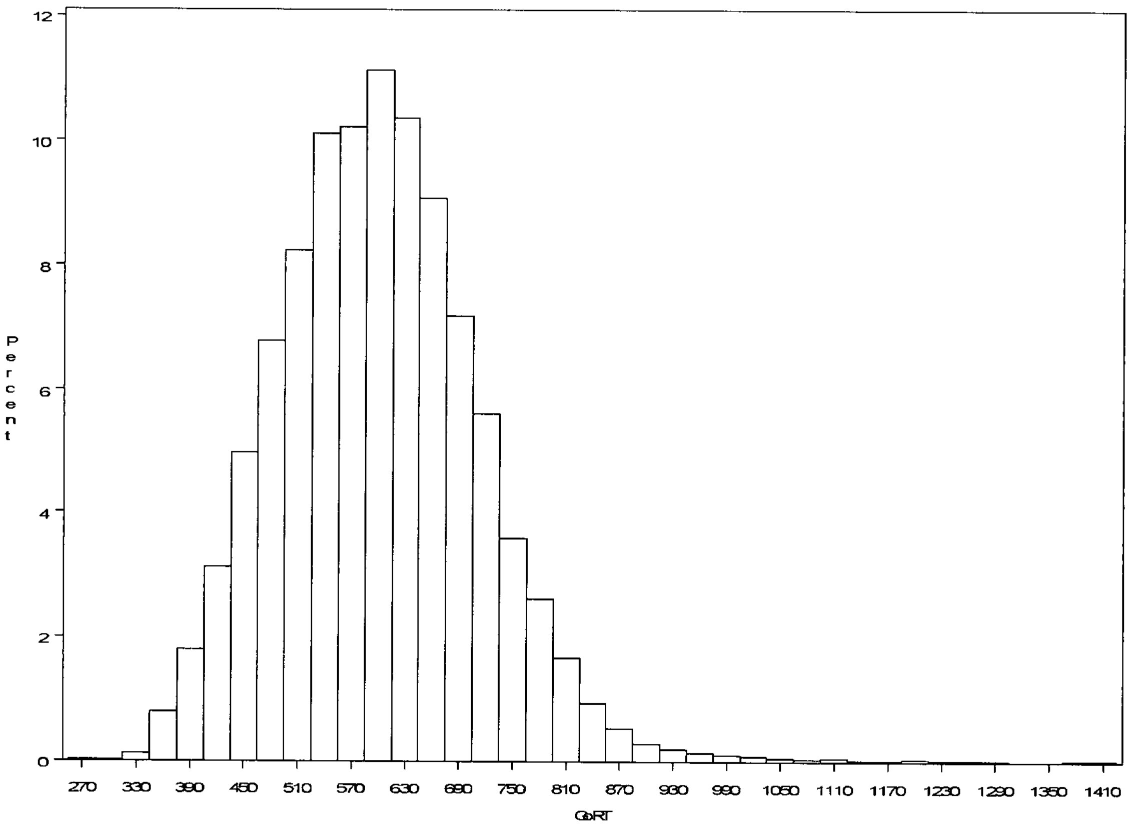

Supplementary Figure 3 Distribution of GoRTSD in sample

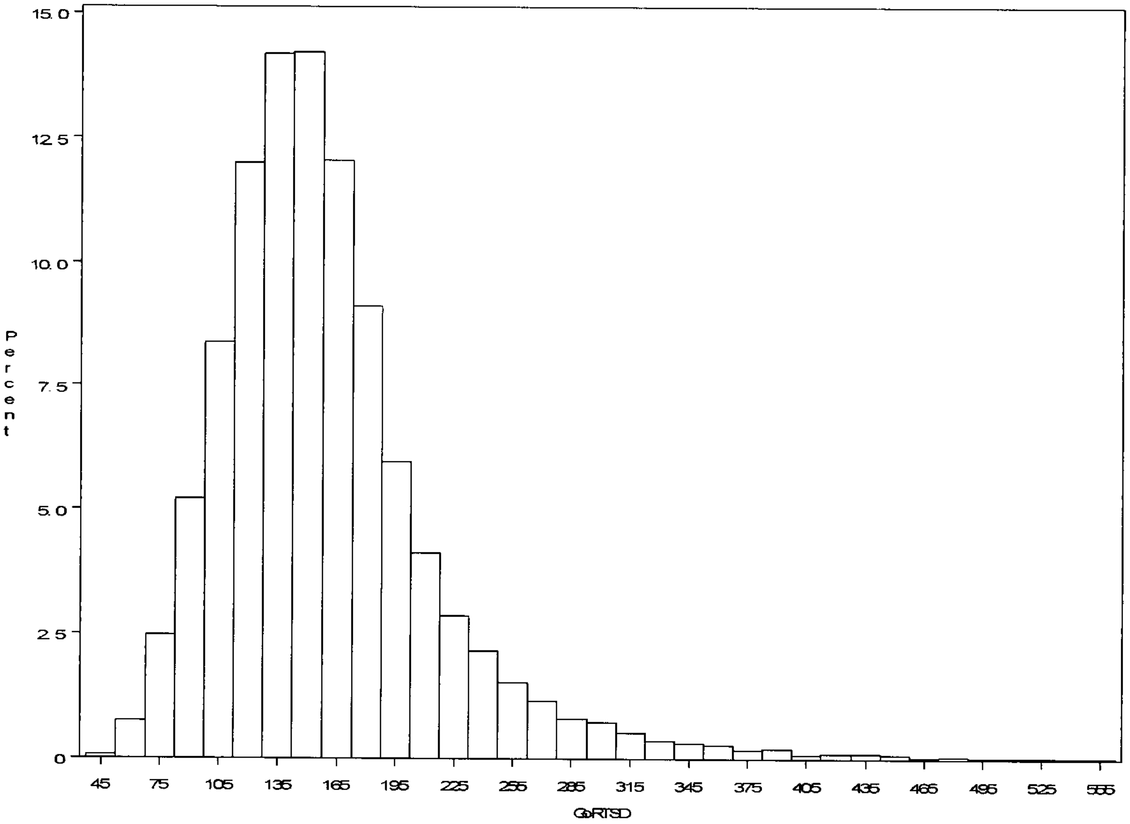

Supplementary Figure 4 Q-Q plot for SSRT

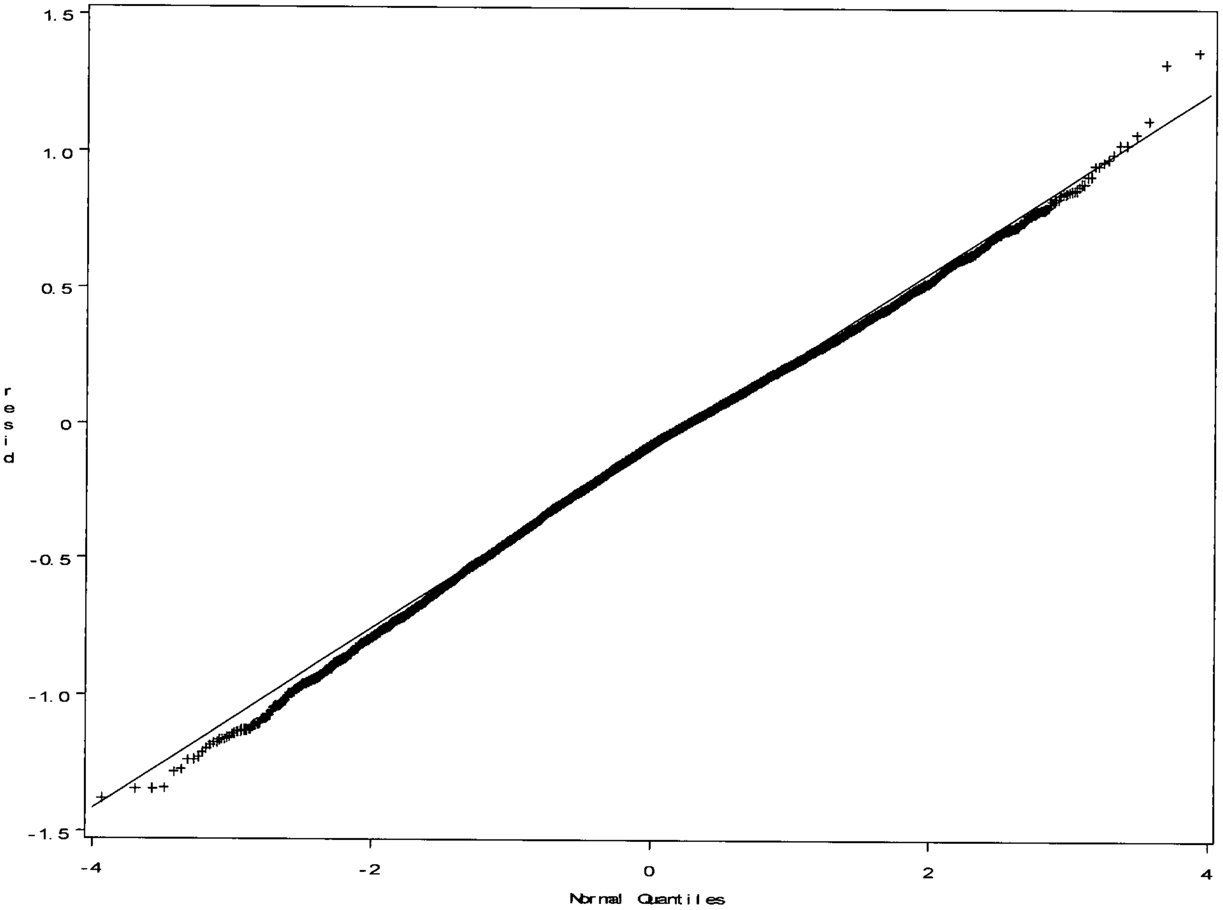

Supplementary Figure 5 Q-Q plot for GoRT

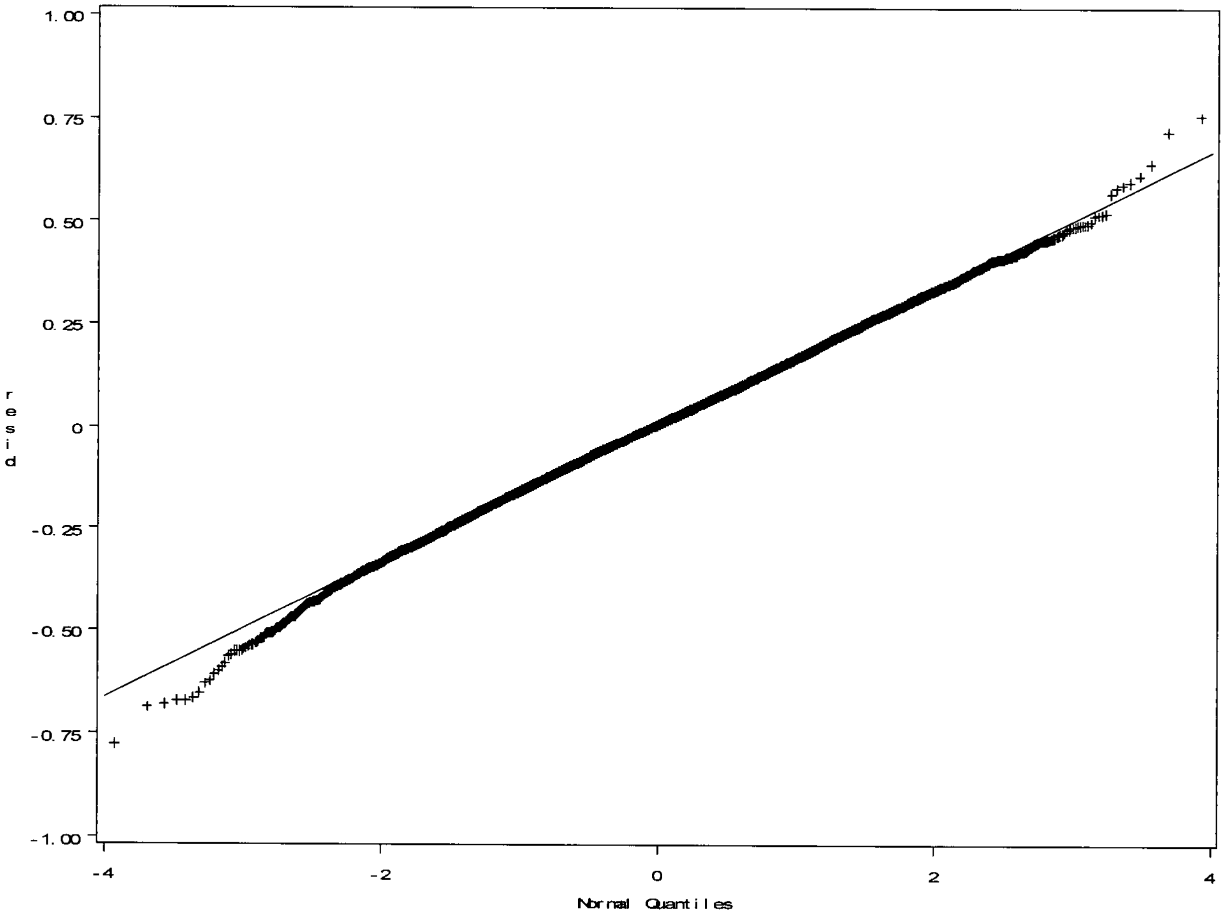

Supplementary Figure 6 Q-Q plot for GoRTSD

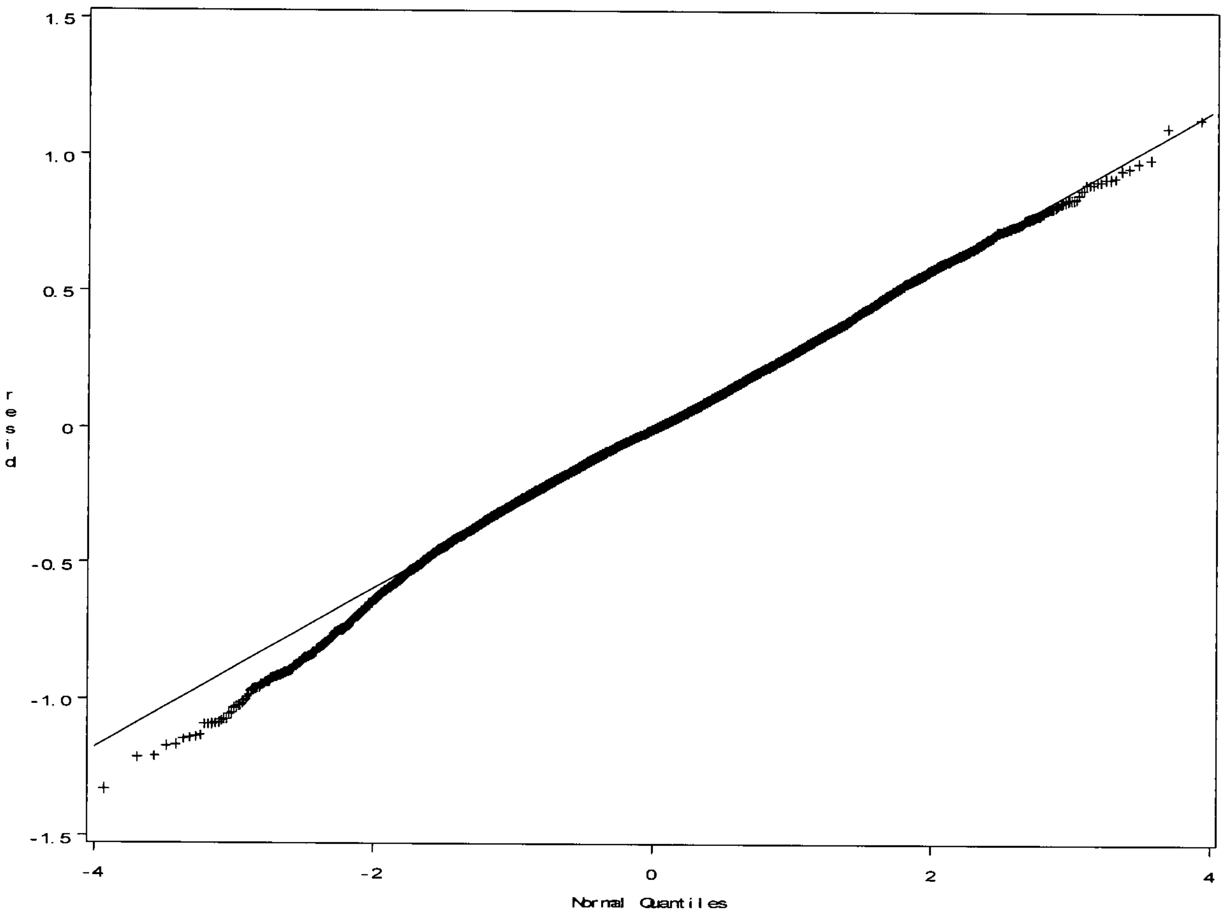

Supplement: Supplementary file 1 — (PDF 42.9 KB) [file 10802_2012_9693_MOESM1_ESM.pdf]
